# Supplementary material for: Evaluation of Endospore-Forming Bacteria for Suppression of Postharvest Decay of Apple Fruit
Source: Microorganisms. 2022 Dec 28;11(1):81. doi: 10.3390/microorganisms11010081 (PMC9862789; doi:10.3390/microorganisms11010081)
Supplement: Supplementary file 1 [file microorganisms-11-00081-s001.zip › microorganisms-2095040-supplementary.pdf]

Supplemental Figure S1

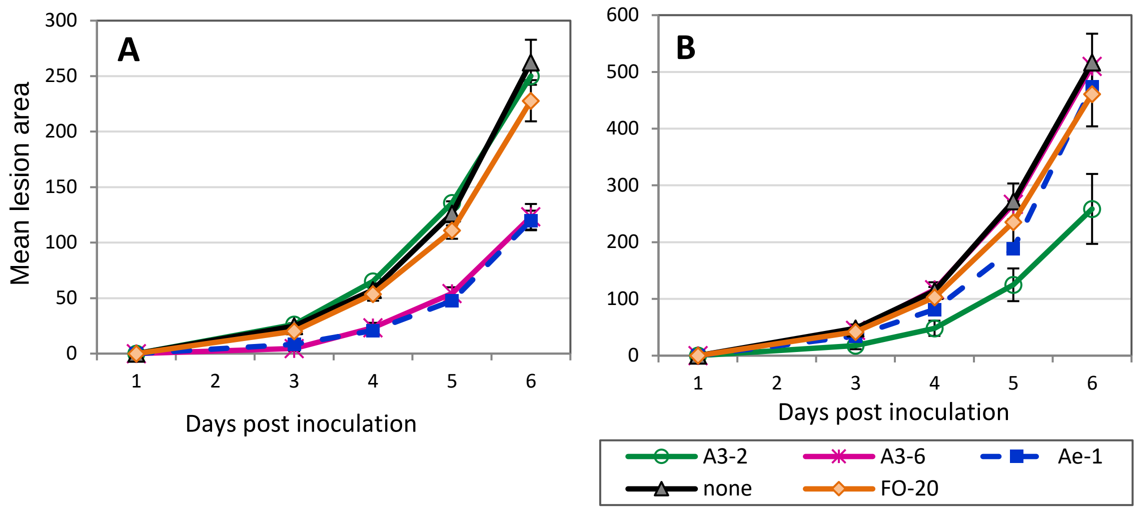

Supplemental Figure S1: Mean lesion area on wounded 'Golden Delicious' fruit inoculated with 10^4 conidia/ml of (**A**) *C. acutatum* and (**B**) *P. expansum*. Error bars represent the standard error of the mean. Bacteria were applied at 10^8 CFU/ml at day 0. Lesion diameters were measured vertically and horizontally across the wound site 3-, 4-, 5- and 6-days post inoculation with the bacteria and pathogen.
